# Myeloid Sarcoma Arising in a Rare Anatomical Location: A Case Report

**DOI:** 10.7759/cureus.104962

**Published:** 2026-03-10

**Authors:** Effrosyni Bompou, Maria M Pitsilka, Dimosthenis Chrysikos, Maria Piagkou, Gerasimos Tsourouflis, Theodore Troupis

**Affiliations:** 1 Department of Surgery, University Hospital of Larissa, Larissa, GRC; 2 Department of Anatomy, National and Kapodistrian University of Athens, Athens, GRC; 3 Department of Anatomy/Department of Oral Surgery, National and Kapodistrian University of Athens, Athens, GRC; 4 School of Medicine, National and Kapodistrian University of Athens, Athens, GRC; 5 2nd Department of Propaedeutic Surgery, Laiko General Hospital, Athens, GRC; 6 Department of Surgery, National and Kapodistrian University of Athens, Athens, GRC

**Keywords:** acute myeloid leukemia, case report, chloroma, extramedullary myeloid tumor, myeloid sarcoma

## Abstract

Myeloid sarcoma (MS) is a rare extramedullary tumor of immature myeloid cells that can occur concurrently with acute myeloid leukemia (AML), precede its onset, or present in isolation. Gastrointestinal involvement is uncommon, and diagnosis is often challenging due to non-specific imaging features and clinical presentation.

We report the case of a 75-year-old patient with newly diagnosed AML who was found to have synchronous cecal wall thickening and an adjacent hypodense lesion on abdominal computed tomography (CT). Due to an impending obstruction, surgery was performed, and the patient underwent ileocecectomy with primary anastomosis. Histopathology revealed extensive infiltration of the intestinal wall by immature hematopoietic cells, immunopositive for leukocyte common antigen (LCA), myeloperoxidase (MPO), and c-Kit, consistent with synchronous MS. Postoperatively, the patient was managed with standard AML therapy (chemotherapy with cytarabine plus an anthracycline was initiated).

MS is difficult to diagnose radiologically and may mimic other malignancies or inflammatory processes. Definitive diagnosis relies on histopathology and immunohistochemistry. No standardized treatment exists; AML-based chemotherapy remains the mainstay, with hematopoietic stem cell transplantation considered for treatment intensification. Surgical intervention is reserved for complications such as obstruction or perforation.

This case underscores the rarity and diagnostic challenge of synchronous MS in AML. Early recognition requires high clinical suspicion, and diagnosis depends on pathology. Further studies are needed to guide optimal management strategies.

## Introduction

Myeloid sarcoma (MS) refers to a tumor formed by myeloid blasts, regardless of their degree of maturation, occurring outside the bone marrow, with diagnosis requiring clear evidence of effacement of the normal tissue structure at the involved site [1]. It has also been referred to in the literature as chloroma or granulocytic sarcoma; however, the term MS is currently the most widely accepted designation.

Although the precise incidence of MS is difficult to determine, a recent retrospective observational cohort study by Goyal et al. suggests that it occurs in approximately 0.8% of patients with acute myeloid leukemia (AML), with a slight male predominance (56%) [2].

According to the largest study based on a US registry, the most frequent sites of involvement are connective and soft tissues (31.3%), followed by the skin and breast (12.3%) and the gastrointestinal tract (10.3%) [2]. Within the gastrointestinal tract, however, involvement of specific anatomical subsites is considerably less common and may represent a particularly rare clinical presentation.

Gastrointestinal MS may also present significant diagnostic challenges, as its clinical and radiologic features can overlap with those of other gastrointestinal malignancies or inflammatory conditions, potentially leading to diagnostic delay.

The management of MS is challenging, as clear guidelines are lacking. Although approximately 70% of patients present with symptoms, aggressive surgical intervention is generally not recommended prior to the initiation of systemic therapy. Local treatments, such as radiotherapy, can be effective, but they are not considered first-line therapy [3]. However, management should be individualized, as the approach depends on the tumor's location and the nature of the symptoms.

In this report, we present a case of MS arising in a rare gastrointestinal location, highlighting the diagnostic considerations and therapeutic approach in this uncommon presentation.

## Case presentation

**Figure 1:**
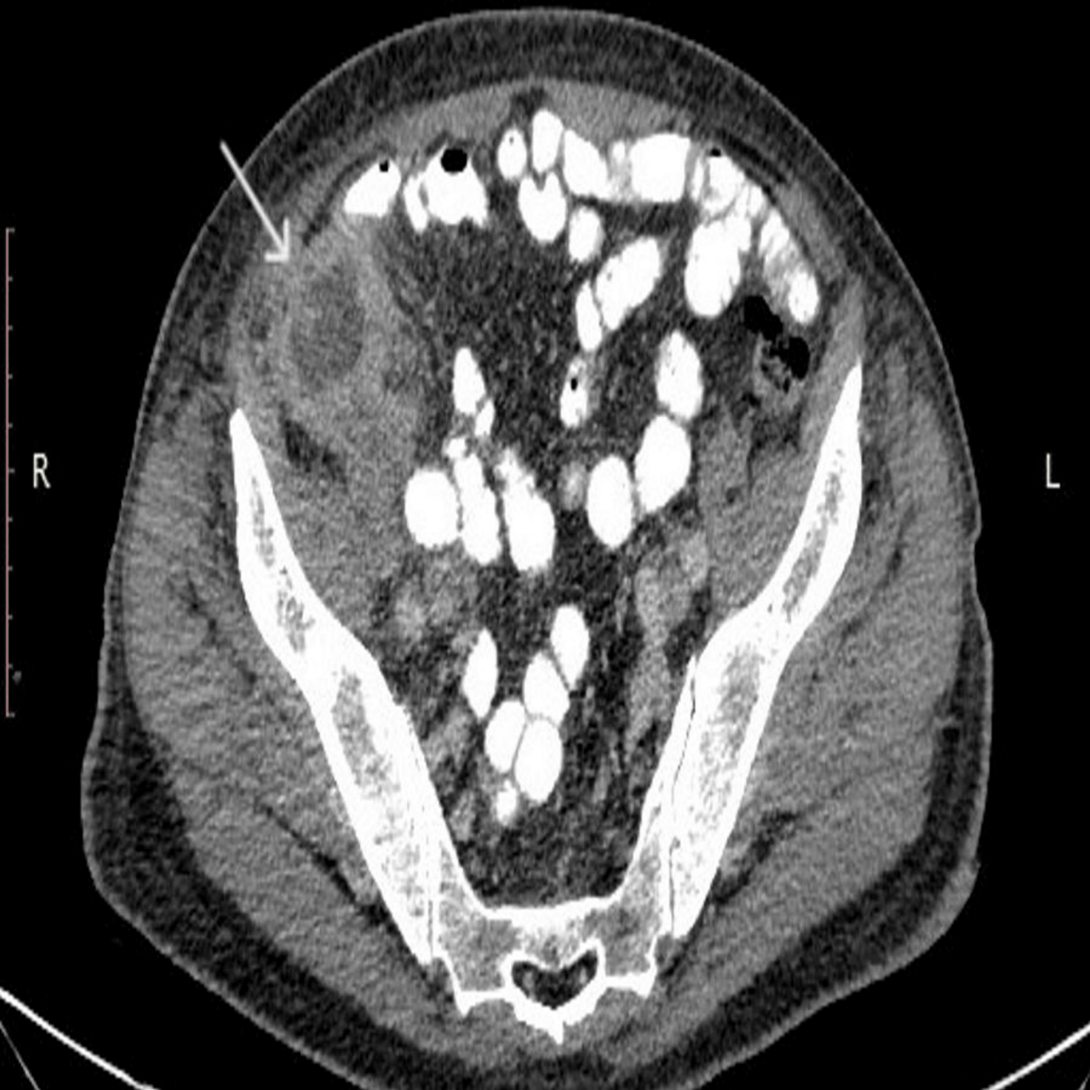
Abdominal CT scan (axial and coronal) showing the lesion CT: computed tomography

**Figure 2:**
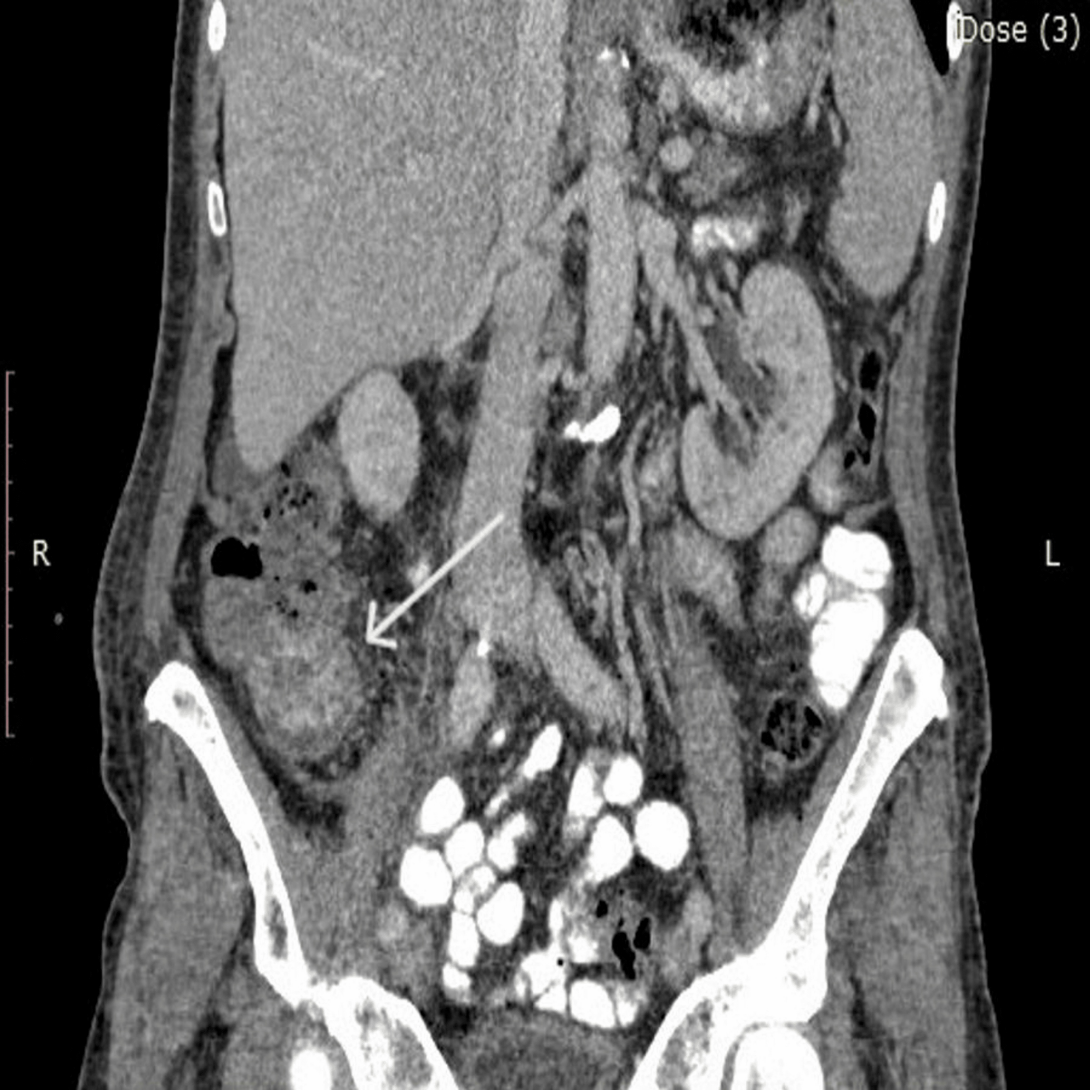
Abdominal CT scan (axial and coronal) showing the lesion CT: computed tomography

**Figure 3:**
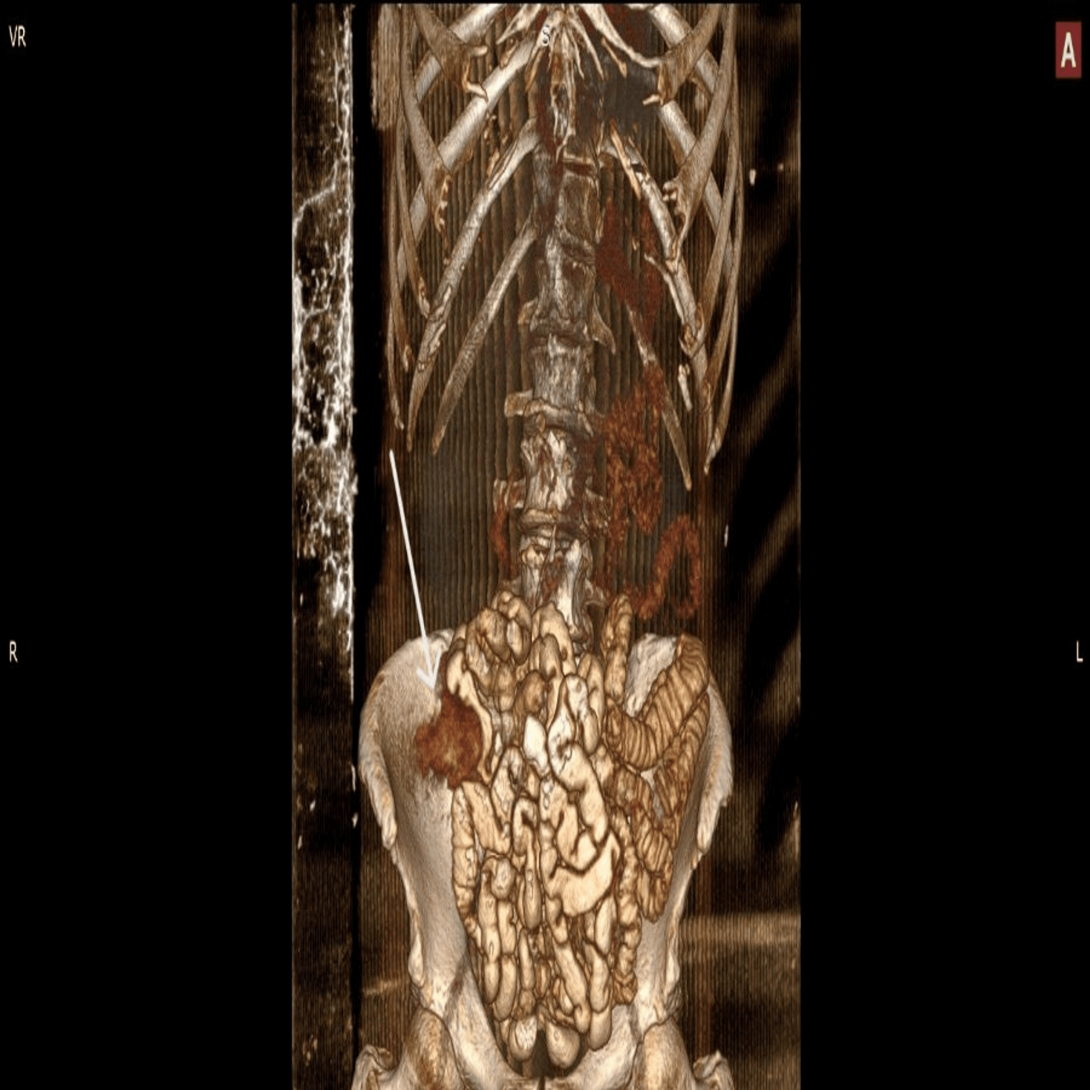
Abdominal CT scan 3D reconstruction showing the lesion CT: computed tomography

A 75-year-old patient with newly diagnosed AML was hospitalized in the Hematology Department. As part of the initial staging, an abdominal computed tomography (CT) scan was performed, which revealed marked thickening of the cecal wall. Adjacent to it, a hypodense likely cystic lesion with a thickened rim was identified, measuring approximately 4.5 cm in maximal transverse diameter and 3.7 cm craniocaudally. There was notable edema of the surrounding mesenteric fat, with regional lymph nodes measuring up to 1.5 cm, as well as fascial thickening (Figure 1, Figure 2, and Figure 3).

These findings were considered most consistent with an inflammatory process predominantly involving the appendix, which, in the clearly visualized segment, demonstrated a luminal diameter of 1.2 cm and mural thickening. However, a mass lesion in the region remained within the differential diagnosis. The patient was therefore transferred to the Surgical Department for further evaluation and management of the identified lesion. The patient's past medical history was unremarkable. He was not on any chronic medication. He had undergone an elective inguinal hernia repair several years earlier and was a former smoker with a 35-pack-year history. He reported no known allergies.

Based on CT findings and the patient's clinical presentation with signs of partial bowel obstruction, he underwent a semi-urgent ileocecectomy with primary intestinal anastomosis. His postoperative course was uneventful, with no surgery-related complications. He demonstrated return of bowel function and was advanced to oral intake on postoperative day 4. From the culture of the peritoneal fluid obtained intraoperatively, *Escherichia coli* and *Pseudomonas aeruginosa* were isolated. Antibiotic therapy was subsequently adjusted according to the susceptibility profile.

The patient was monitored daily by both the surgical and hematology teams. Serial laboratory testing demonstrated a progressive increase in white blood cell count, reported as lymphocyte-predominant on automated differential; in the context of AML, these cells likely represent circulating myeloid blasts. This was accompanied by a decline in platelet count and hemoglobin levels (Table 1). Consequently, on postoperative day 5, a diagnosis was established, and following the recommendation of the hematologists, hydroxyurea was initiated at a dose of 500 mg twice daily.

**Table 1:** Laboratory test results WBC: white blood cells; HGB: hemoglobin; HCT: hematocrit; PLT: platelets; LY%: lymphocyte percentage; CRP: C-reactive protein

	Preoperative	1st postoperative day	5th postoperative day	Normal range
WBC (×10³/µL)	43000	58800	135400	4500-10500
HGB (g/dL)	9.8	10	8.4	14-18
HCT (%)	29.9	31	28	42-52
PLT (×10³/µL)	76000	63000	28000	140000-440000
LY% (%)	98.2	99.4	99.2	40-70
CRP (mg/L)	24	35	13.8	0.01-0.5

On postoperative day 6, the patient developed a fever of up to 38°C. He was re-evaluated by the hematology team and, on postoperative day 7, was transferred to the Hematology Department to initiate treatment for the AML.

Histopathological examination revealed extensive infiltration of the intestinal wall by medium-sized immature hematopoietic cells, characterized by irregular nuclei and scant, eccentric cytoplasm. Immunohistochemical staining demonstrated positivity for leukocyte common antigen (LCA), myeloperoxidase (MPO), and c-Kit. A subset of these cells also showed positivity for CD15. The cells were negative for CD34, CD20, CD79a, CD3, CD138, and pan-cytokeratins. Based on these findings and the patient's clinical history, the cellular infiltrate is consistent with the involvement of the intestinal wall and perienteric tissues by immature hematopoietic cells in the context of AML. Notably, the appendix was surrounded by this infiltrate but did not show evidence of an active, primary inflammatory process. Additionally, two small adenomas with moderate dysplasia were identified in the cecal region. No adenocarcinoma was observed.

## Discussion

MS is an extremely rare clinical entity. It can appear in several ways, occurring at the same time as leukemia, exactly like in our case, emerging before leukemia develops, appearing together with other myelodysplastic syndromes, or presenting as an isolated mass [4]. Gastrointestinal involvement is uncommon, accounting for approximately 6.5% of reported cases, and involvement of the colon, particularly the cecum, is considered especially rare [5].

Unfortunately, the diagnosis can be difficult due to the fact that CT imaging features are fairly non-specific, making MS difficult to differentiate from other entities such as non-Hodgkin lymphoma, small round-cell tumors like neuroendocrine or carcinoid tumors, as well as metastatic lesions to the intestine [6]. In our case, the imaging findings were also non-specific, initially raising suspicion for a primary intestinal malignancy. Moreover, primary or isolated MS can be difficult to diagnose because it may occur without simultaneous bone marrow infiltration or other systemic manifestations. To minimize diagnostic errors, incorporating myeloid markers like MPO and CD117 into the immunohistochemical panel for lymphoma evaluation is advised [7]. In our case, inclusion of MPO and c-Kit (CD117) in the immunohistochemical panel was crucial for establishing the diagnosis. Definitive diagnosis of MS relies on histopathology supported by immunohistochemical staining, which helps distinguish it from other possible conditions.

Due to the fact that randomized prospective studies are lacking, no standardized treatment approach for MS has been established. At present, the preferred therapy for patients with either isolated MS or MS occurring alongside AML is to use standard AML-based chemotherapy regimens [8]. A combination of cytarabine and anthracycline agents is indicated. Moreover, autologous and allogeneic hematopoietic stem cell transplantation (HSCT) are generally viewed as approaches to intensify treatment [9].

Extramedullary disease often indicates aggressive biology and may associate with poorer outcomes, although data are heterogeneous. Surgical resection is not routinely recommended since it has not been shown to improve survival. Nevertheless, it may be indicated for complications such as obstruction or perforation [10]. In the present case, surgical intervention was performed due to clinical signs of evolving bowel obstruction before the definitive diagnosis was established.

## Conclusions

This case report highlights the rarity of synchronous MS in a patient with AML, particularly when located in the cecum. Early diagnosis may be challenging and requires a high index of clinical and radiologic suspicion, while definitive diagnosis relies on histopathologic and immunohistochemical evaluation. Due to the rarity of this entity and the absence of controlled studies, a standardized treatment approach has not been established. Systemic chemotherapy remains the cornerstone of treatment, whereas the role of surgical intervention is generally limited to the management of complications. Optimal management requires close multidisciplinary collaboration among radiologists, surgeons, and hematologists. Further prospective multicenter studies are needed to better define optimal management strategies for these patients.
